# Comparative analysis between patients undergoing Gastric Bypass and Sleeve Gastroplasty in a private hospital in Sao Luis-MA^1^

**DOI:** 10.1590/s0102-865020200030000007

**Published:** 2020-05-20

**Authors:** Rodrigo Lira Sousa Lima, Eduardo Jose Silva Gomes de Oliveira, Emanuel Cabral Pereira, Lucas da Silva Costa, Thiago Sousa Dourado, José Aparecido Valadão, Roclides Castro Lima, Giuliano Peixoto Campelo, Roger Moura de Brito, Caio Márcio Barros de Oliveira, Ed Carlos Rey Moura, Plinio da Cunha Leal

**Affiliations:** I Graduate student, Departamento de Medicina I, Universidade Federal do Maranhão (UFMA), Sao Luis-MA, Brazil. Conception and design of the study; acquisition, analysis and interpretation of data; statistics analysis, manuscript preparation and writing.; II Graduate student, Departamento de Medicina I, UFMA, Sao Luis-MA, Brazil. Manuscript preparation and writing.; III Graduate student, Departamento de Medicina I, UFMA, Sao Luis-MA, Brazil. Conception and design of the study, manuscript preparation and writing.; IV Graduate student, Departamento de Medicina I, UFMA, Sao Luis-MA, Brazil. Conception and design of the study; acquisition, analysis and interpretation of data; manuscript preparation and writing.; V PhD, Departamento de Medicina II, UFMA, Sao Luis-MA, Brazil. Manuscript preparation and writing, critical revision.; VI MD, Departamento de Cirurgia, Hospital São Domingos, Sao Luis-MA, Brazil. Manuscript preparation and writing, critical revision.; VII PhD, Departamento de Medicina I, UFMA, Sao Luis-MA, Brazil. Analysis and interpretation of data, manuscript preparation and writing, critical revision.; VIII PhD, Departamento de Medicina I, UFMA, Sao Luis-MA, Brazil. Scientific and intellectual content of the study, analysis and interpretation of data, manuscript preparation, critical revision.

**Keywords:** Obesity, Gastroplasty, Gastric Bypass

## Abstract

**Purpose:**

To compare the satisfaction levels about the surgery and anesthesia management, and to analyze the postoperative outcomes of patients undergoing Gastric Bypass and Sleeve Gastroplasty surgeries in a private hospital in Sao Luís-MA.

**Methods:**

The sample consisted of patients undergoing Bypass and Sleeve bariatric surgeries from August 2018 to August 2019, who were in the range of 18 and 70 years old and had not used drugs or presented cardiac arrhythmias, dilated cardiomyopathy, and conduction disorder heart. Data were collected from the evaluation forms and recorded in a form with closed questions.

**Results:**

Most patients were female (Bypass – 56% and Sleeve – 67.4%) and aged between 30 and 39 years old (Bypass – 32% and Sleeve – 55.8%). Information (Bypass – 92% and Sleeve – 86.1%) was the highest satisfaction index found. Sleepiness in the immediate postoperative period (Bypass – 92% and Sleeve – 93%) was the main side effect. There were no postoperative complications in patients between the two types of surgery.

**Conclusions:**

Patients submitted to Bypass and Sleeve were completely satisfied with the perioperative management. There was no statistically significant difference when comparing adverse effects between the techniques.

## Introduction

Obesity is considered an increasingly common disease, the prevalence of which is already reaching epidemic proportions and has serious social and psychological consequences. Studies underline that overweight and obesity will reach levels of 89% in men and 85% in women by 2030^1^.

Regarding its characteristics, obesity is a condition that affects individuals of all age groups, social classes, genders, and races. It has a multifactorial etiology, with hereditary correlation, since children of obese parents are 80 to 90% more likely to become obese^2^ ^,^ ^3^.

Obesity is usually classified by body mass index (BMI), which is the internationally accepted standard method accepted by researchers, with weight in kg divided by the square of height in meters - equal to or greater than 30kg/m² ^4^. Regarding severity, WHO proposes the classification in degrees, being specified as grade I obesity, when the BMI is between 30 and 34.9 kg / m²; grade II obesity, when BMI is between 35 and 39.9 kg / m²; and grade III obesity when BMI is above 40 kg / m² ^5^. The prevalence of obesity has doubled in the last 30 decades and is also considered the first epidemic of the twentieth century^6^.

The presence of obesity results in several metabolic complications, which can trigger issues related to physical, psychological and social aspects. Thus, the disease rarely acts in isolation, aggravating or causing other risks such as diabetes mellitus (especially type 2), various types of cancer, hypertension, and osteoarthritis, which may negatively affect the life quality and longevity^7^.

Regarding the treatment of obesity, some options cover the clinical and surgical aspects. The clinical treatment of obesity includes changes in behavior, such as physical activity stimulation, dietary changes with restriction of calorie intake, in addition to psychotherapy and the use of some types of medications^8^. However, morbidly obese patients do not respond satisfactorily to the clinical treatment of obesity, since most of them cannot maintain weight reduction for more than two years^9^. In such cases, bariatric surgery is recommended, as it is a more effective treatment method in terms of extension and weight reduction^10^. For several years, Roux-en-Y Gastric Bypass (RGBY) was considered the gold standard procedure for bariatric techniques and the most commonly performed form of surgery. However, in recent years, vertical gastrectomy has proved to be a highly effective and simpler procedure, and this has led to its increased popularity^11^.

Despite being an efficient approach in the treatment of obesity, bariatric surgeries have several possible postoperative complications, which can be divided into early and late ones. The early ones generate significant morbidity and an increase in hospitalization costs^12^. However, complications are rare and mortality related to bariatric surgeries remains below 1%^13^.

Patient satisfaction is an important factor in the outcome of health care and the evaluation of the services quality in surgical interventions^14^ ^,^ ^15^. Satisfaction is a subjective and multidimensional concept, influenced by cultural, sociodemographic, physical, mental and emotional factors^14^ ^,^ ^16^ ^,^ ^17^. With an increasing focus when evaluated as an indicator of quality, it can reveal the patients’ perception of the outcome of the intervention and their overall experience, providing information about the hospital capacity to provide a quality service that meets the patient's expectations^15^ ^,^ ^17^ ^,^ ^18^. Despite this setting, not many studies are found in the literature that specifically assess the satisfaction levels with the service provided in bariatric surgeries.

Thus, this study aimed to compare the satisfaction levels regarding the surgery and anesthesia management and to analyze the postoperative outcomes of patients undergoing Gastric Bypass and Sleeve Gastroplasty surgeries in a private hospital in Sao Luis-MA.

## Methods

### Study design

This study was approved by the Research Ethics Committee of São Domingos Hospital (number 90023118500005085). This is a descriptive, prospective, observational and comparative study with a quantitative approach from August 2018 to August 2019, at the Center for Bariatric and Metabolic Surgery of São Domingos Hospital (Sao Luis-MA).

### Sample

The sample consisted of patients undergoing Bypass and Sleeve bariatric surgeries from August 2018 to August 2019, who were between 18 and 70 years old and had not used drugs and did not present cardiac arrhythmias, dilated cardiomyopathy or cardiac conduction disorder. The bariatric surgery technique was chosen following the institution treatment protocol, which takes into consideration the body mass index, associated comorbidities and the presence or not of reflux.

Considering studying about 10 patients/month and estimating a confidence interval of 95%, a margin of error of 5%, it was estimated to study 92 patients in the period. Assuming the losses by the exclusion criteria, the final sample was 68 patients.

### Instruments for data collection

#### Sociodemographic, clinical characteristics and satisfaction level form

The volunteers were interviewed by the researcher to fill out a sociodemographic form with information on age, gender, race/skin color, municipality of residence, marital status, and academic degree. Surgery time, drugs used and time to wake up were evaluated in the intraoperative period.

The satisfaction levels about the surgery and anesthetic management were analyzed through a patient-reported outcome questionnaire in Brazilian Portuguese designed for the study and composed of six items addressing the following aspects of the patient in the perioperative period: Attention, Information, Privacy, Waiting Time, Pain Control and Discomfort. The items were graded according to the Likert scale, with the options Very Dissatisfied, Dissatisfied, Satisfied, Very Satisfied and Completely Satisfied, scoring from 1 to 5, with a minimum of 5 points to a maximum of 30 points. Patients answered the questionnaire at the immediate post-surgery (time 0), 2h, 6h, 18h, 24h, 7 days and 1 month after surgery.

In the postoperative outcomes, the surgery and anesthesia side effects of the complications were evaluated, if present or not, including nausea, vomiting, sleepiness and dizziness. Complications included bleeding, fistulas, stenosis, obstructions, and others. It was also analyzed the return of feeding, elimination of flats, walking. These variables were analyzed in the immediate postoperative after waking up (time zero), 2h, 6h, 12h, 24h, 7 days and 1 month after surgery. The return of sexual function was evaluated 7 days and 1 month after surgery.

The presence of pain was assessed at rest and in motion in the immediate postoperative period when the patient woke up (time zero), 2h, 6h, 18h, 24h, 7 days and 1 month after surgery.

The data required in the first 24 hours after surgery were collected in the patients’ hospital room. Within 7 days and 1 month after the surgery, the data were collected by telephone.

The anesthesia used in the study was through the total intravenous anesthesia technique with induction by propofol 2 mg/kg, fentanyl 3-5 mg/kg and rocuronium 0.6 g/kg. For maintenance, propofol and remifentanil were used targeting the Bispectral index (BIS) 40-60. Prior to extubation, dipyrone 2g, dexamethasone 10mg, ondansetron 8mg, morphine 0.1mg/kg and sugammadex 4mg/kg were administered.

### Statistical analysis

Data were first tabulated through Microsoft Excel 13 software. Then statistical tests were performed using Stata 14 software. Categorical variables were organized into frequency tables for each period and analyzed using Pearson's chi-square test or if indicated, by Fisher's test. Continuous variables were described in measures of central tendency and variance and then compared by Student's t-test. The level of statistical significance used for all tests was 5% (p < 0.05).

## Result

A total of 68 subjects participated in the study. Sociodemographic data were described in Table 1. There was a predominance of females (56% Bypass and 67.6% Sleeve) in both types of surgery (p = 0.345) and the most prevalent age group was between 30 and 39 years (p = 0.078), although without statistical difference. Regarding academic degree, there was no statistically significant difference (p = 0.478). In Bypass, most patients had completed higher education (76%), and the same result was found for the Sleeve (58.13%). Regarding the degree of obesity, there was a quantitative difference between the two surgical procedures. Most (69.8%) of the patients who underwent sleeve surgery were in preoperative obesity grade 2. For those who underwent bypass surgery, 64% had Grade 3 obesity before surgery (p = 0.00).

**Table 1:** Epidemiological data and degree of obesity in patients undergoing Bypass or Sleeve.

		Bypass		Sleeve		p
		N	%	N	%	
Gender						
	Male	11	44.0	14	32.6	0.345
	Female	14	56.0	29	67.4	
Age range						
	10-19 anos	1	4.0	0	0.0	0.078
	20-29 anos	5	20.0	8	18.6	
	30-39 anos	8	32.0	24	55.8	
	40-49 anos	5	20.0	9	20.9	
	50-59 anos	4	16.0	2	4.7	
	60-69 anos	2	8.0	0	0.0	
Race/Color						
	White	21	84.00	34	79.06	0.312
	Dun	4	16.00	4	9.30	
	Black			5	11.64	
Marital Status						
	Married	16	64.00	32	74.41	0.346
	Single	5	20.00	9	20.93	
	Live together	2	8.00	1	2.33	
	Divorced	2	8.00	1	2.33	
Obesity						
	Overweight	0	0.0	1	2.3	0.00
	Degree 1	4	16.0	7	16.3	
	Degree 2	5	20.0	30	69.8	
	Degree 3	16	64.0	5	11.6	
**Total**		**25**	**100,0**	**43**	**100,0**	

Test Chi – Quadrado

Regarding the time of surgery, Bypass had a longer time, with an average of 115.76 minutes, and Sleeve had an average of 109.93 minutes. However, in this variable, there was no statistically significant difference (p = 0.0907) (Table 2).

**Table 2:** Procedure time of patients undergoing Bypass or Sleeve.

	Bypass		Sleeve		Total		p
	Average	±SD*	Average	±SD	Average	±SD	
Time	115.76	20.71	106.5349	22.25	109.93	22.00	0.0907

Test T de Student

* SD = Standard Deviation

The patients analyzed were completely satisfied in both groups. It is noteworthy that there was a statistical difference (p=0,025) concerning health care attention. In the Bypass group, 88% of patients said they were completely satisfied with the attention of health professionals, whereas 95.4% of those in the Sleeve group had the same answer. Regarding other satisfaction indicators, there was no statistical difference; even though, it is worth noting that most patients felt well informed, with their privacy respected, had a reasonable waiting time for surgery, good pain control and mild discomfort in the postoperative period (Table 3).

**Table 3:** Satisfaction indicators of patients undergoing Bypass or Sleeve.

		Bypass		Sleeve		p
		N	%	N	%	
Attention						
	3	0	0.0	2	4.7	0.025
	4	3	12.0	0	0.0	
	5	22	88.0	41	95.4	
Information						
	2	1	4.0	0	0.0	0.367
	3	0	0.0	2	4.7	
	4	1	4.0	4	9.3	
	5	23	92.0	37	86.1	
Privacy						0.648
	3	0	0.0	1	2.3	
	4	1	4.0	3	7.0	
	5	24	96.0	39	90.7	
Waiting time						
	1	0	0.0	1	2.3	0.913
	2	1	4.0	3	7.0	
	3	3	12.0	4	9.3	
	4	4	16.0	6	14.0	
	5	17	68.0	29	67.4	
Pain Control						
	3	2	8.0	2	4.8	0.495
	4	1	4.0	5	11.9	
	5	22	88.0	35	83.3	
Discomfort						
	3	2	8.0	2		0.494
	4	1	4.0	5	11.6	
	5	22	88.0	36	83.7	
**Total**		**25**	**100.0**	**43**	**100.0**	

Square Chi Test

In postoperative pain, T0 (time zero) was the time in which there was the most complaint of rest pain in patients undergoing Bypass (56%), with no statistical difference between groups (p = 0.132) (Table 4).

**Table 4:** Postoperative pain assessment of patients undergoing Bypass or Sleeve.

		ByPass		Sleeve		p
		N	%	N	%	
Resting pain T0						
	No	11	44.00	27	62.79	0.132
	Yes	14	56.00	16	37.21	
Resting pain 2h						
	No	15	60.00	25	58.14	0.881
	Yes	10	40.00	18	41.86	
Resting pain 6h						
	No	20	80.00	35	81.40	0.888
	Yes	5	20.00	8	18.60	
Resting pain 18h						
	No	24	96.00	39	90.70	0.419
	Yes	1	4.00	4	9.30	
Resting pain 24h						
	No	24	96.00	41	97.62	0.706
	Yes	1	4.00	1	2.38	
Resting pain 7 days						
	No	24	100.00	41	95.35	0.283
	Yes	0	0.00	2	4.65	
Resting pain 30 days						
	No	24	96.00	43	100.00	0.186
	Yes	1	4.00	0	0.00	
Pain in motion T0						
	No	25	100.00	43	100.00	
	Yes	0	0	0	0	
Pain in motion 2h						
	No	25	100.00	43	100.00	
	Yes	0	0	0	0	
Pain in motion 6h						
	No	25	100.00	42	97.67	0.442
	Yes	0	0.00	1	2.33	
Pain in motion 18h						
	No	24	96.00	43	100.00	0.186
	Yes	1	4.00	0	0.00	
Pain in motion 24h						
	No	25	100.00	43	100.00	
	Yes	0	0	0	0	
Pain in motion 7 days						
	No	25	100.00	42	97.67	0.442
	Yes	0	0.00	1	2.33	
Pain in motion 30 days						
	No	25	100.00	43	100.00	
	Yes	0	0	0	0	
**Total**		**25**	**100.00**	**43**	**100.00**	

Square Chi Test

By evaluating the side effects (nausea, vomiting, dizziness, sleepiness, and others), there was a higher frequency of sleepiness, followed by nausea; sleepiness (p = 0.876) was found in 92% of patients who underwent Bypass and 93% of those who underwent Sleeve. It is noteworthy that there was a statistically significant difference (p = 0.038) in the nausea side effect 6 hours after surgery. At the time, 20% of patients who underwent bypass reported feeling nauseous and 46.5% of those who underwent sleeve reported this side effect.

There is also a statistically significant difference (p = 0.024) in the immediate postoperative dizziness side effect (time zero). Of the patients who underwent bypass, 68% reported this side effect, while those who underwent sleeve corresponded to 90.7%.

In terms of postoperative evolution, we evaluated the return of food, the elimination of flatus, the return of ambulation and sexual activity. From 18 hours after surgery, patients of both types of gastroplasty (Bypass, 76%; Sleeve, 69.8%) began to restore fluid intake, with no statistically significant difference between groups (p = 0.581).

In regards to flatus, 24 hours after surgery, the majority of patients (Bypass 72%; Sleeve 86.1%) had already confirmed that they had eliminated flatus, with no statistically significant difference (p = 0.156). Concerning the return to ambulation, after 6 hours of the surgical procedure, patients were already able to walk through the hospital corridors (Bypass 84%; Sleeve 79.1%), also without difference between groups (p = 0.618).

Analyzing the return of sexual activity after 30 days of the surgical procedure, most patients had sexual intercourse in the period (Bypass 84%; Sleeve 72.1%), but no statistically significant difference was found (p = 0.264). In addition, in regards to early postoperative complications, no patient who underwent any of the two types of bariatric surgery in this study had complications (bleeding, fistula and others) until the first month after surgery.

## Discussion

There was a predominance of females among the patients evaluated for both Bypass and Sleeve. This result corroborates those studies conducted in the Northeast region of Brazil^19^, Brazil^20^ ^,^ ^21^ and international studies^22^ ^,^ ^23^. Epidemiological data showed, in Brazil, a higher prevalence of obesity in women compared to men, with a difference of 0.6% between both genders^19^.

In addition to epidemiological evidence, unlike women, men resort to surgical treatment when their physical activities are compromised^24^. What could also justify the predominance of women in surgical treatment for obesity is the change in physical appearance, for aesthetic motivation or beauty standards formalized by society and the media^19^.

An age range of 30 to 39 years was more frequent in the present study, similar to a survey in which the average age was 34 years. However, in several studies, the mean age group is in the 40's range^26^ ^-^ ^28^. The proportion of overweight and obese adults increased between 1980 and 2013 from 28.8% to 36.9% in men^29^; besides, by 2030, 86% of adults in the United States will have obesity^30^. Thus, the average age of studies converges with the increased prevalence of this disease in the adult population.

Regarding the degree of obesity found in the present study, there was a divergence from another study^31^, in which the majority of patients (59.6%) evaluated were in Grade 3. In two other studies, the mean BMI value is within the Class 3 Obesity classification^32^ ^,^ ^33^. However, the authors state that there are benefits of surgery for individuals with grades of obesity 1 and 2, or even overweight when associated with comorbidities occurrence^31^. Thus, patients with a lower grade of obesity and with associated comorbidities are being submitted to bariatric surgery earlier.

Information, communication, respect, and patient care are recognized for being among the most important factors about patient care^34^ ^-^ ^36^. In this study, we achieved good results with the items in the satisfaction questionnaire; however, one limitation was not using a fully validated questionnaire, as it consisted of only several items and not a multidimensional evaluation^37^. Larger studies on patient satisfaction are necessary to improve the quality of the service, identify factors that can be improved and bring greater satisfaction to patients undergoing this type of surgery.

In the analysis of postoperative side effects, other studies^19^ ^,^ ^38^ had similar results, since nausea and vomiting were listed as one of the most frequent alterations found, besides hair loss and nutritional deficiency. As seen in the present study, as well as those found in the literature, postoperative changes are frequent, requiring health professionals to be prepared to make early diagnosis and intervention, to attenuate the impact on patients’ health and quality of life, thus achieving better results^19^ ^,^ ^39^.

Regarding immediate complications, wound infection, bleeding, deep vein thrombosis and pulmonary embolism are the most common ones^40^. In our study, from the immediate postoperative period up to the first 30 days, there was no report of any unfavorable outcome (bleeding, fistula, and others) in the patients analyzed in both groups. A study shows/reveals that mortality in a 30-day postoperative period after bariatric surgery is less than 1%^12^.

One of the important factors that have a direct impact on quality of life is sexual function and desire. In this study, the return of sexual function was quite significant in both surgical procedures, but it was more frequent in patients who underwent Bypass surgery (84%) compared to those who underwent Sleeve surgery (72.1%). Similar data were found in another study^41^, in which 44.68% reported increased sexual interest after bariatric surgery.

Although not statistically relevant between the two surgical procedures, pain in this study was found more prevalent in patients undergoing the Bypass procedure in the first hours after surgery. A similar percentage was found in another study^42^, with the incidence of pain in the Post Anesthetic Recovery Room (PACU) being 52.5% (n = 63); 36.11% reported severe pain (defined as a score of 8 to 10 on the numeric scale). The incidence of postoperative pain after bariatric surgery in patients undergoing general anesthesia is a factor to be considered when choosing intraoperative anesthetics^42^.

## Conclusions

A relevant rate of bariatric surgery was observed, especially in the female public. When comparing adverse effects between Gastric Bypass and Sleeve Gastroplasty, there was no statistically significant difference. Patients submitted to Bypass and Sleeve were completely satisfied with the perioperative management. They reported feeling well informed, and having their privacy respected, a reasonable waiting time for surgery, good pain control, and mild post-operative discomfort. Postoperative complications, when they appeared, had a lower frequency than in literature. However, due to the obesity epidemic and the increasing frequency of bariatric surgical procedures, the encouragement of new studies on patient satisfaction levels with perioperative management can improve the quality of the service offered and benefit this portion of society.

## References

[1] Keaver L, Weeber L, Dee A, Shiely F, Marsh T, Balanda K, Perry IJ (2013). Application of the UK foresight obesity model in Ireland: the health and economic consequences of projected obesity trends in Ireland. PLoS One.

[2] Karim MA, Ahmed J, Arneil C, Ali A (2013). Utilization of hospital services by obese patients before and after bariatric surgery. Surg Today.

[3] Wannamacherw L (2016). Obesidade como fator de risco para morbidade e mortalidade: evidências sobre o manejo com medidas não medicamentosas. PAHO.

[4] Engin A (2017). The definition and prevalence of obesity and metabolic syndrome. Adv Exp Med Biol.

[5] WHO (2000). Obesity: preventing and managing the global epidemic. Report of a WHO consultation.

[6] Dias PC, Henriques P, Anjos LA, Burlandy L (2017). Obesity and public policies: the Brazilian government's definitions and strategies. Cad Saúde Pública.

[7] Meldrum DR, Morris MA, Gambone JC (2017). Obesity pandemic: causes, consequences, and solutions—but do we have the will?. Fert Steril.

[8] Teixeira PI, Carraca EV, Marques MM (2015). Successful behavior change in obesity interventions in adults: a systematic review of self-regulation mediators. BMC Med.

[9] Arterburn DE, Courcolas AP (2014). Bariatric Surgery for obesity and metabolic conditions in adults. BMJ.

[10] Mun EC, Blackburn GL, Matthews JB (2001). Current status of medical and surgical therapy for obesity. Gastroenterology.

[11] Buchwald H, Williams SE (2016). Bariatric surgery worldwide. Obes Surg.

[12] Jammah AA (2015). Endocrine and metabolic complications after bariatric surgery. Saudi J Gastroenterol.

[13] Flum DR, Belle SH, Longitudinal Assessment of Bariatric Surgery (LABS) Consortium (2009). Perioperative safety in the longitudinal assessment of bariatric surgery. N Engl J Med.

[14] Shnaider I, Chung F (2006). Outcomes in day surgery. Curr Opin Anaesthesiol.

[15] Heidegger T, Saal D, Nübling M (2013). Patient satisfaction with anaesthesia - Part 1: satisfaction as part of outcome - and what satisfies patients. Anaesthesia.

[16] Auquier P, Pernoud N, Bruder N, Simeoni MC, Auffray JP, Colavolpe C, François G, Gouin F, Manelli JC, Martin C, Sapin C, Blache JL (2005). Development and validation of a perioperative satisfaction questionnaire. Anesthesiology.

[17] Nakahira J, Sawai T, Ishio J, Nakano S, Minami T (2019). Factors associated with poor satisfaction with anesthesia in patients who had previous surgery: a retrospective study. Anesth Pain Med.

[18] Lyu H, Wick EC, Housman M, Freischlag JA, akary MA (2013). Patient satisfaction as a possible indicator of quality surgical care. JAMA Surg.

[19] Castanha CR, Castanha AR, Belo GQ, Lacerda RM, Vilar L (2018). Evaluation of quality of life, weight loss and comorbidities of patients undergoing bariatric surgery. Rev Col Bras Cir.

[20] Driscoll S, Gregory DM, Fardy JM, Twells LK (2016). Long-term health-related quality of life in bariatric surgery patients: a systematic review and meta-analysis. Obesity (Silver Spring).

[21] Duarte MI, Bassitt DP, Azevedo OC, Waisberg J, Yamaguchi N, Pinto PE (2014). Impact on quality of life, weight loss and comorbidities: a study comparing the biliopancreatic diversion with duodenal switch and the banded Roux-en-Y gastric bypass. Arq Gastroenterol.

[22] Puzziferri N, Austrheim IT, Wolfe BM, Wilson SE, Nguyen NT (2006). Three-year follow-up of a prospective randomized trial comparing laparoscopic versus open gastric bypass. Ann Surg.

[23] Schauer PR, Ikramuddin S, Gourash W, Ramanathan R, Luketich J (2000). Outcomes after laparoscopic Roux-en-Y gastric bypass for morbid obesity. Ann Surg.

[24] de Siqueira LT, Wanderley MSO, da Silva RA, Silva AAP, Lima JL, Ferraz AAB (2018). A screening study of potential carcinogen biomarkers after surgical treatment of obesity. Obes Surg.

[25] Milone M, Lupoli R, Maietta P, Di Minno A, Bianco P, Ambrosino P, Coretti G, Milone F, Di Minno MN, Musella M (2015). Lipid profile changes in patients undergoing bariatric surgery: a comparative study between sleeve gastrectomy and mini-gastric bypass. Int J Surg.

[26] Kansou G, Lechaux D, Delarue J, Badic B, Le Gall M, Guillerm S, Bail JP, Thereaux J (2016). Laparoscopic sleeve gastrectomy versus laparoscopic mini gastric bypass: One year outcomes. Int J Surg.

[27] Musella M, Apers J, Rheinwalt K, Ribeiro R, Manno E, Greco F, Cierny M, Milone M, Di Stefano C, Guler S, van Lessen IM, Guerra A, Maglio MN, Bonfanti R, Novotna R, Coretti G, Piazza L (2015). Efficacy of bariatric surgery in type 2 Diabetes mellitus remission: the role of mini gastric bypass/one anastomosis gastric bypass and sleeve gastrectomy at 1 year of follow-up. A European survey. Obes Surg.

[28] Ortega E, Morínigo R, Flores L, Moize V, Rios M, Lacy AM, Vidal J (2012). Predictive factors of excess body weight loss 1 year after laparoscopic bariatric surgery. Surg Endosc.

[29] Ng M, Fleming T, Robinson M, Thomson B, Graetz N, Margono C (2014). Global, regional, and national prevalence of overweight and obesity in children and adults during 1980–2013: a systematic analysis for the Global Burden of Disease Study 2013. Lancet.

[30] Ginter E, Simko V (2014). Becoming overweight: is there a health risk?. Bratisl Lek Listy.

[31] Steyer NH, Oliveira MC, Gouvêa MRF, Echer IC, Lucena AF (2016). Clinical profile, nursing diagnoses and nursing care for postoperative bariatric surgery patients. Rev Gaúcha Enf.

[32] Salminen P, Helmiö M, Ovaska J, Juuti A, Leivonen M, Peromaa-Haavisto P, Hurme S, Soinio M, Nuutila P, Victorzon M (2018). Effect of laparoscopic sleeve gastrectomy vs laparoscopic Roux-en-Y gastric bypass on weight loss at 5 years among patients with morbid obesity. JAMA.

[33] Azagury D, Mokhtari T, Garcia L, Rosas U, Garg T, Rivas H, Morton J (2019). Heterogeneity of weight loss after gastric bypass, sleeve gastrectomy, and adjustable gastric banding. Surgery.

[34] Heidegger T, Saal D, Nübling M (2013). Patient satisfaction with anaesthesia - Part 1: Satisfaction as part of outcome - and what satisfies patients. Anaesthesia.

[35] Heidegger T, Husemann Y, Nuebling M, Morf D, Sieber T, Huth A, Germann R, Innerhofer P, Faserl A, Schubert C, Geibinger C, Flückiger K, Coi T, Kreienbühl G (2002). Patient satisfaction with anaesthesia care: development of a psychometric questionnaire and benchmarking among six hospitals in Switzerland and Austria. Br J of Anaesth.

[36] Mui WC, Chang CM, Cheng KF, Lee TY, Ng KO, Tsao KR, Hwang FM (2011). Development and validation of the questionnaire of satisfaction with perioperative anesthetic care for general and regional anesthesia in taiwanese patients. Anesthesiology.

[37] Berning V, Laupheimer M, Nübling M, Heidegger T (2017). Influence of quality of recovery on patient satisfaction with anaesthesia and surgery: a prospective observational cohort study. Anaesthesia.

[38] Duarte MI, Bassitt DP, Azevedo OC, Waisberg J, Yamaguchi N, Pinto PE (2014). Impact on quality of life, weight loss and comorbidities: a study comparing the biliopancreatic diversion with duodenal switch and the banded Roux-en-Y gastric bypass. Arq Gastroenterol.

[39] Stoll A, Rosin L, Dias M, Marquiotti B, Gugelmin G, Stoll G (2016). Early Postoperative complications in Roux-en-Y gastric bypass. ABCD Arq Bras Cir Dig (São Paulo).

[40] Schauer P, Ikramuddin S, Hamad G, Gourash W (2003). The learning curve for laparoscopic Roux-en-Y gastric bypass is 100 cases. Surg Endosc.

[41] Mendes G, Vargas G (2017). Quality of life after vertical gastrectomy evaluated by the Baros questionnaire. ABCD Arq Bras Cir Dig.

[42] Silva LM, Silveira SQ, Abib ACV, Nunes WPM, Mittermayer O, Oliveira DR (2018). Comparative analysis of remifentanil versus dexmedetomidine in the incidence of pain in a post – anesthesia care unit after bariatric. BRJP São Paulo.

